# Uncovering the Potential Link Between Polychlorinated Biphenyls and Cardiovascular Diseases: A Comprehensive Analysis

**DOI:** 10.3390/toxics13020071

**Published:** 2025-01-22

**Authors:** Jingyu Liu, Qiuli Shan, Yang Yang, Wenxing He

**Affiliations:** 1 College of Biological Science and Technology, University of Jinan, Jinan 250022, China; jy18739485166@163.com; 2 College of Marine Life Science, Ocean University of China, Qingdao 266003, China; 21240613013@stu.ouc.edu.cn

**Keywords:** PCBs, CVDs, meta-analysis, systematic review, public health

## Abstract

Background: A family of persistent organic pollutants, known as polychlorinated biphenyls (PCBs), are extensively found in the environment and may be harmful to the cardiovascular system. Systematic reviews and meta-analyses are required to thoroughly evaluate the association between PCB exposure and cardiovascular disease (CVDs), despite the fact that studies on the subject have produced inconsistent results. Objective: The purpose of this study was to investigate the relationship between PCBs exposure and cardiovascular disease risk in order to provide more conclusive data to promote public health actions. Methods: The studies that met the inclusion criteria were screened out using the databases PubMed, Web of Science, ScienceDirect, and Cochrane Library. The comprehensive effect size (OR) was calculated using the random-effects model; the study’s heterogeneity was analyzed using I^2^ statistics; the major reasons of heterogeneity were identified using subgroup analysis; and publication bias graphically was measured using the Egger’s test. Results: A meta-analysis of 11 studies revealed that total PCBs (OR = 1.56, 95% CI: 1.20–1.75), non-dioxin-like PCBs (NDL-PCBs) (OR = 1.33, 95% CI: 1.15–1.53), and dioxin-like PCBs (DL-PCBs) (OR = 1.31, 95% CI: 1.10–1.57) were all found to be positively associated with the risk of cardiovascular disease. Subgroup analysis revealed that study type, biomaterials, and literature quality were the most significant drivers of variation. Furthermore, certain PCB homologues, such as non-dioxin-like (NDL)-PCB153 and dioxin-like (DL)-PCB118, are highly related with cardiovascular disease. Conclusions: According to this meta-analysis, exposure to PCBs may increase the risk of cardiovascular disease. Notwithstanding major drawbacks, our results emphasize the significance of lowering exposure to PCBs and offering a solid theoretical basis for public health initiatives.

## 1. Introduction

Cardiovascular diseases (CVDs) are disorders that affect the heart and blood vessels, including coronary heart disease, myocardial infarction, stroke, and arteriosclerosis [1]. These situations interrupt normal blood circulation and oxygen flow, which may cause organ damage [2]. CVDs pose a significant global health threat and are among the main causes of death [3]. They can lead to serious complications such as myocardial infarction and thrombosis, significantly impairing patients’ quality of life and posing potentially life-threatening risks [4]. Obesity, hypertension, diabetes, high cholesterol and triglyceride levels, chronic psychological stress, genetic susceptibility, and bad lifestyle choices have all been identified as risk factors for CVDs [5,6]. Persistent organic pollutants (POPs) have received more attention lately. Studies show that POP exposure in the environment plays a major role in the development and progression of CVDs [7].

POPs are natural or manmade organic contaminants that remain in environmental media such as air, water, soil, and living organisms for long periods, posing major hazards to the environment and human health [8]. PCBs are a common class of POPs. According to studies, PCBs are persistent, bioaccumulative, semi-volatile, and highly poisonous [9]. These compounds possess the potential to induce permanent damage to the neurological, endocrine, and reproductive systems. As lipophilic substances, they can disrupt intracellular lipid metabolism, thereby affecting metabolic and endocrine functions. There is a strong link between PCBs and chronic metabolic disorders, such as diabetes, obesity, CVDs, and other chronic diseases, according to recent epidemiological research [10,11]. PCBs have been linked to metabolic syndrome (MetS), which is linked to the type and homologue of PCBs, according to a meta-analysis [12]. The findings of another meta-analysis indicate that PCBs, particularly DL-PCBs, constitute a significant risk factor for the development of hypertension [13].

The number of articles and reports discussing the link between PCBs and CVDs has significantly increased in recent years, raising public awareness of PCBs. Individual studies produced inconsistent or contradictory results, and there has been some research on the association between PCBs and CVDs, but it has not been systematically integrated as of late. A 2016 cohort study of men who were Swedish citizens in 1997 discovered a link between dietary PCB exposure and an increased risk of myocardial infarction [14]. On the other hand, a cross-sectional examination in 2020 revealed a common association between coronary artery calcification and PCB exposure not seen in the diet [15]. A 2021 study linked six serum PCB homologues to CVD prevalence [16]. In contrast, Laura Deen et al. found no such association [17]. Therefore, more research is required to validate the link between PCBs and CVDs.

The objectives we set in doing this meta-analysis were to better understand the variations in PCBs levels across individuals with and without CVDs and to assess the correlation between PCBs and CVDs. Additionally, we performed a subgroup analysis of research design, PCB type, and article quality, three possible impacting factors.

## 2. Materials and Methods

### 2.1. Methods

This study was conducted in accordance with the PRISMA 2020 (Preferred Reporting Items for Systematic Reviews and Meta-Analyses) [18] statement and the MOOSE (Meta-analyses of Observational Studies in Epidemiology) guidelines [19]. This study has been registered as PROSPERO, Registration number CRD42024563475. The following is the Population, Exposure, Comparator, and Outcome (PECO) [20] for this topic: Population (P): General population, including those exposed to PCBs. Exposure (E): Exposure to or increased exposure to PCBs as indicated by the results of human blood, serum, or adipose tissue tests. Comparator (C): Unexposed or less exposed to PCBs (if applicable) or lowest PCB exposure group. Outcome (O): Occurrence of CVDs.

### 2.2. Exposure and Outcome Measures

Assessments were conducted individually for PCBs, dioxin-like (DL) PCBs, non-dioxin-like (NDL) PCBs, and selected individual congeners. The primary outcome was incidence or prevalence of CVDs.

### 2.3. Literature Sources and Search Methodologies

Our research team looked through PubMed, ScienceDirect, Web of Science, and the Cochrane Library for pertinent literature. Database retrieval period began on 1 March 2024. Due to variations in search methods across different websites, the keywords used for searches may differ. Please refer to Supplementary Materials for specific search strategies.

### 2.4. Criteria for Study Selection

Studies that fulfilled the following criteria were considered epidemiological: (1) any demographic or subject group; (2) case-control, cross-sectional, and queuing study designs (the literature that is included needs to address the connection between cardiovascular illnesses and homologues of polychlorinated biphenyls); (3) biological samples (blood, serum, or adipose tissue) for the purpose of measuring PCBs directly or indirectly through consumption of PCB-estimating foods; (4) the incidence or prevalence of cardiovascular disease, together with the related 95% confidence intervals (CI) for the hazard ratio (HR), relative risk ratio (RR), and odds ratio (OR); (5) English should be the language used for the included books; (6) original research should be the genre of the incorporated literature.

### 2.5. Criteria for Study Exclusion

Our exclusion criteria were: (1) Studies that are not research-focused on PCBs and CVDs; (2) literature encompassing both animal and in vitro experiments; (3) the language of literature is not English; (4) topics for literature include reviews, analyses, abstracts, and so on; (5) studies lacking valid data in their findings.

### 2.6. Quality Evaluation

For the cohort and case-control studies included in the literature, the Newcastle–Ottawa Quality-Assessment Scale (NOS) was used for evaluation [21]; for cross-sectional studies, a modified NOS scale [22] was applied.

### 2.7. Data Extraction

We will extract the following information from all included studies: authors, publication year, design of the study, location, study population, study period, sample size, gender ratio, age distribution, biological substances, types of PCBs, methods of PCBs measurement, outcomes, and main findings.

### 2.8. Result Analysis

The effect sizes and confidence intervals between PCBs and CVDs will be calculated using the raw data from the literature. The relationship between PCBs and CVDs will be investigated through the synthesis of effect sizes. The heterogeneity of the combined effect was examined using the Q test. A significant level of heterogeneity is indicated by an effect size of the Q test (I^2^) larger than 50%, at which point the random-effects model ought to be applied. A fixed-effect model should be used when the heterogeneity is deemed low [23] and the I^2^ value is less than 50%.

Subgroup analysis will be used to investigate the sources of heterogeneity when they are high. Utilizing the funnel plot and Egger test method, the publication bias of the included literature was quantified. To determine how each study affected the total effect size, we conducted sensitivity analyses. After removing one study per round, we ran the meta-analysis. Using stratified analyses, potential causes of heterogeneity related to the quality of the literature, research design, or PCB type were evaluated. The amount of possible confounding (unmeasured, unknown, OR residual) was estimated using the total PCBs measured with OR as the effect, and the e values were provided.

A smaller e value indicates that less unmeasurable confounding is needed to explain the impact estimate, whereas a greater e value indicates the necessity for substantial unmeasurable confounding. Stata MP14 (521.14.0.366) and Review Manager 5.3 (5.3.5.0) were used for the statistical analysis.

## 3. Result

### 3.1. Screening Process

The Cochrane Library, Web of Science, ScienceDirect, and PubMed were the sources of 749 papers that we retrieved. Following a literature search, two researchers (Jingyu Liu and Yang Yang) separately chose the literature. For any objections, the third reviewer (Qiuli Shan) provides additional feedback.

**Figure 1:**
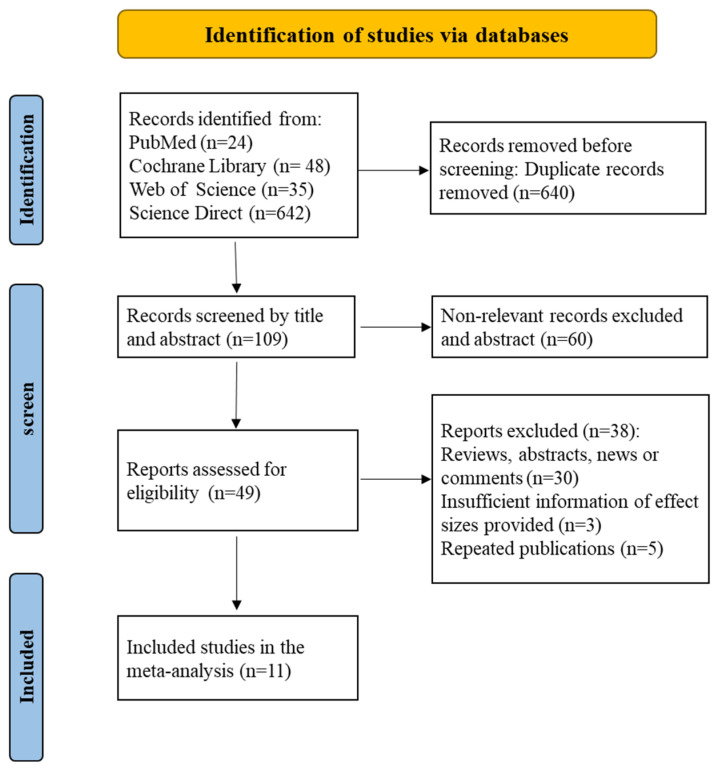
A literature screening flow chart identified a total of 11 articles.

Six hundred forty articles were initially removed based on our inclusion and exclusion criteria after reviewing the titles and abstracts. Following the completion of the literature extraction, the articles that did not meet the requirements and those for which the complete text could not be retrieved were rejected individually by two researchers. After reviewing the full text of the remaining publications using the inclusion and exclusion criteria, a total of 11 papers were included in this analysis, with no articles eliminated due to a lack of complete text (Figure 1).

### 3.2. Research Characteristics

The characteristics of the included studies are presented in Table 1. This systematic review comprised seven cohort studies, three case-control studies, and one cross-sectional research. Five investigations were undertaken in Sweden, two in Spain, and one each in Denmark, Italy, China, and the Republic of Korea. Considering five studies including more than 300 participants and six studies involving more than 1300 participants, the study population was generally big. Eight of the studies included high-exposure subgroups, and three examined the overall population. The majority of research calculates exposure by adding up all of the PCB homologues. Nine studies measured PCB concentrations in serum or plasma, one study measured PCB concentrations in adipose tissue, and one study tested PCB concentrations in lipids. These findings provide insight into the methodologies used to estimate PCB exposure. Nine of the nine studies that assessed PCBs in blood or serum did so as a ratio of total lipids to PCB serum levels (standardized). Almost all studies controlled for age (*n* = 10), smoking habits (*n* = 10), education (*n* = 9), and body mass index (*n* = 9), and most also adjusted for alcohol consumption (*n* = 8), physical activity (*n* = 7), sex (*n* = 7), and sex (*n* = 7). Some studies also adjusted for triglycerides or cholesterol (*n* = 8), family history of genetic disease (*n* = 5), and diabetes (*n* = 4) (Supplemental Materials Table S1).

### 3.3. Comprehensive Analysis

**Figure 2:**
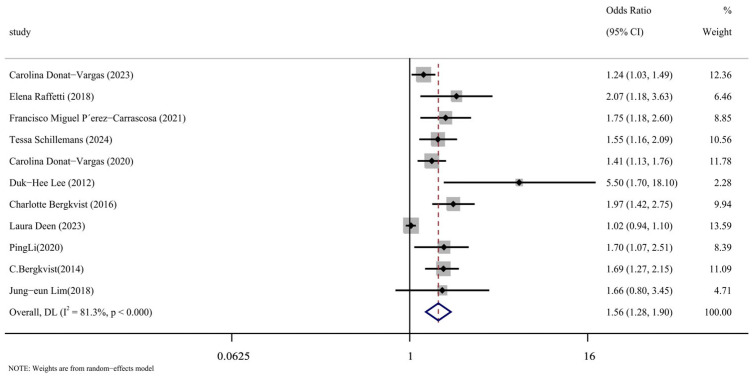
Forest plot of overall analysis of PCBs and CVDs [14,15,16,17,24,25,26,27,28,29,30].

We carefully read the 11 included articles [14,15,16,17,24,25,26,27,28,29,30], extracted the effect indicators of PCBs and CVDs, combined them for meta-analysis, and the results were represented by forest maps, as shown in Figure 2. Through data extraction and analysis, a summary of PCBs and CVDs was produced from the article. When the data were merged, they showed a strong correlation between PCB exposure and the risk of cardiovascular disease (OR = 1.56, 95% CI: 1.28, 1.90, *p* < 0.001). The next section will address the reasons for the significant heterogeneity in this analysis (I^2^ = 81.3%), as indicated by the meta-analysis.

**Figure 3:**
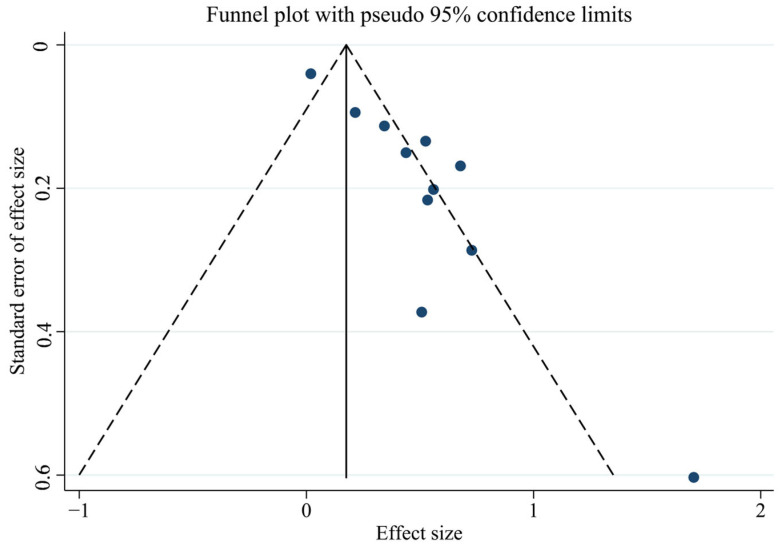
Funnel plot of total PCBs and CVDs overall analysis.

**Figure 4:**
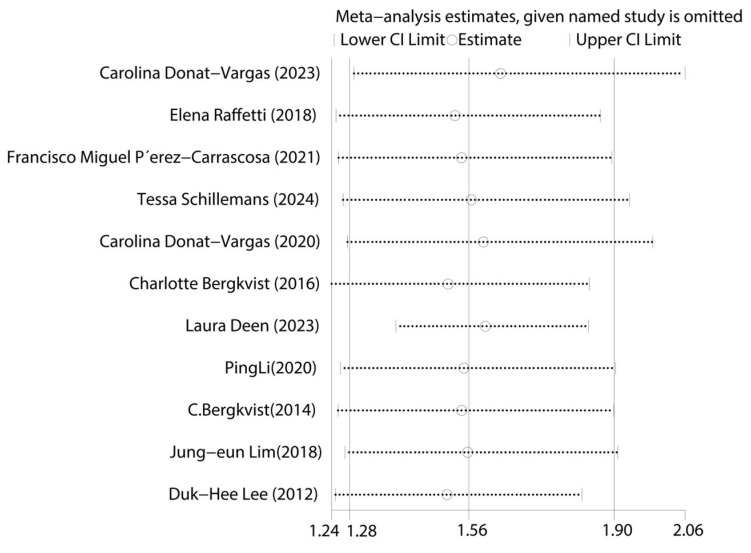
Graph of sensitivity analysis results for the overall analysis of total PCBs and CVDs [14,15,16,17,24,25,26,27,28,29,30].

First, we tested for publication bias using the collected literature. The findings were plotted as a funnel plot in Figure 3, suggesting that the meta-analysis may have some publication bias. To investigate the possibility of publication bias, we used the Egger test on the compiled literature. The test results are displayed in Supplemental Materials Table S2. There is publication bias in the included literature, as indicated by the *p*-value of 0.001. Subsequently, sensitivity analysis was performed on the included studies, and the results are displayed in Figure 4, which demonstrates the robustness of the findings. Consequently, in order to investigate the reasons for heterogeneity, we performed a subgroup analysis.

#### 3.3.1. Subgroup Analysis of Literature Quality

**Figure 5:**
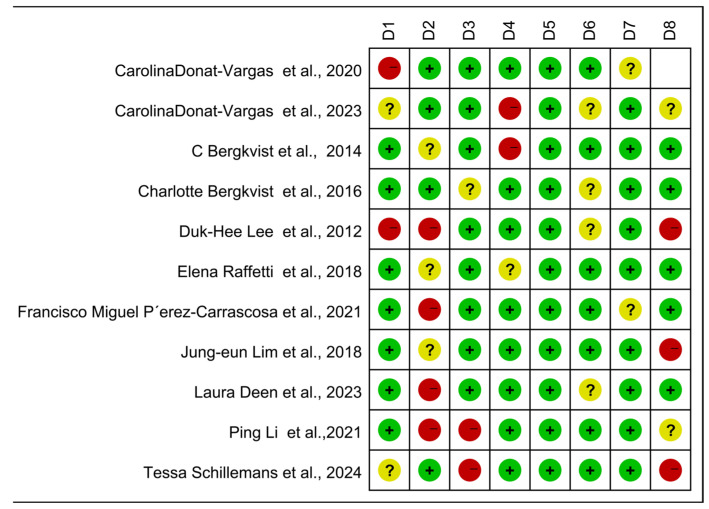
Newcastle–Ottawa Scale (NOS)—Quality assessment. The quality of the studies was evaluated using the NOS. D1–D8 represent NOS items for case-control and cohort studies, while D1–D7 represent adapted NOS items for cross-sectional studies. + for low risk, − for high risk, ? Unclear representation [14,15,16,17,24,25,26,27,28,29,30].

**Figure 6:**
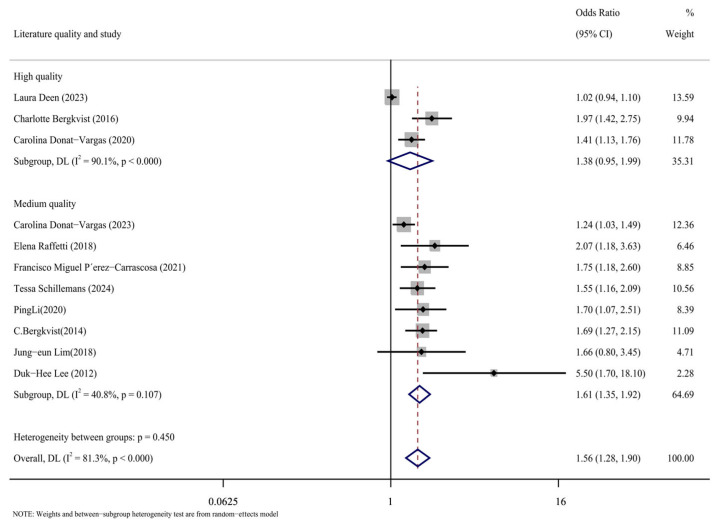
Forest plots with literature quality as subgroup [14,15,16,17,24,25,26,27,28,29,30].

The case-control, cohort, and cross-sectional studies were assessed separately, and the quality of the 11 included publications was assessed using the NOS scoring technique (Figure 5). It can be seen from the figure that there are three kinds of literature of high quality [14,15,17] and eight kinds of literature of medium quality [16,24,25,26,27,28,29,30]. The analysis results are shown in Figure 6, including the group of high quality (OR = 1.38, 95% CI: 0.95, 1.99, *p* < 0.001, I^2^ = 90.1%) and the medium-quality group (OR = 1.61, 95% CI: 1.35, 1.92, *p* = 0.107, I^2^ = 40.8%). The results demonstrate that, whereas the medium-quality group has minimal variability, the high-quality group has significant heterogeneity. According to this analysis, one of the factors contributing to the high consistency of comprehensive results is the high caliber of the literature.

#### 3.3.2. Subgroup Analysis Based on the Kind of Biomaterials Was Conducted

**Figure 7:**
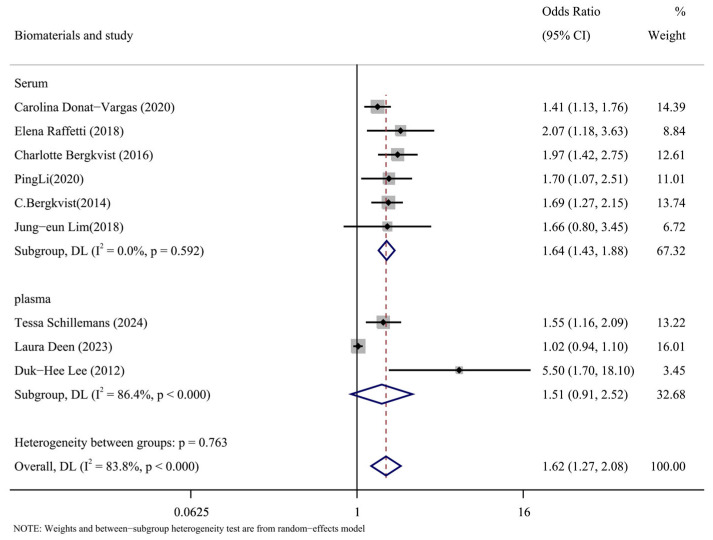
Forest plots of PCBs and CVDs with biomaterial as a subgroup [14,15,17,24,25,26,27,28,30].

The types of biomaterials included in the literature were subgroups analyzed by serum and plasma. The results of the subgroup analysis are shown in Figure 7. The results in the serum group were as follows: (OR = 1.64, 95% CI: 1.43, 1.88, *p* = 0.592, I^2^ = 0%). The results of plasma group were as follows: (OR = 1.51, 95% CI: 0.91, 2.52, *p* < 0.001, I^2^ = 86.4%). According to the data, the plasma group had a significant degree of heterogeneity compared to the serum group’s low level. It is possible to argue that one of the causes of the significant heterogeneity of the overall results was the type of biomaterial called plasma.

#### 3.3.3. Subgroup Analysis Took out Based on the Type of Study

**Figure 8:**
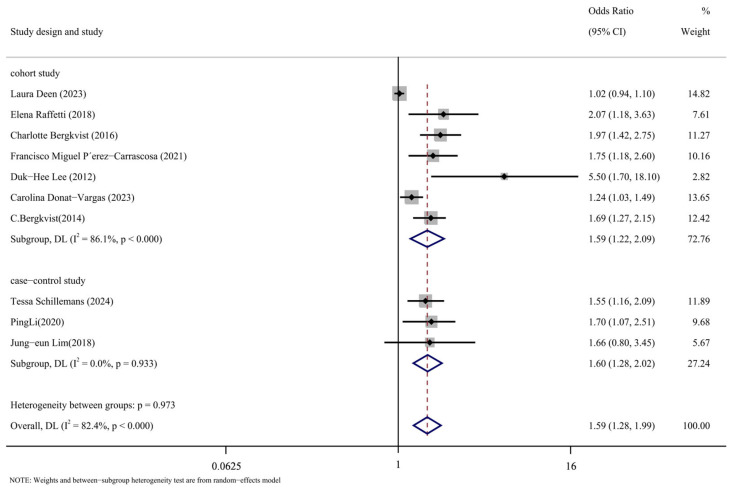
Forest map with research method as subgroup [14,16,17,24,25,26,27,28,29,30].

Subgroup analysis was conducted in accordance with the study’s design. Figure 8 displays the findings of the analysis. The following are the cohort study’s findings: (OR = 1.59, 95% CI: 1.22, 2.09, *p* < 0.001, I^2^ = 86.1%); The case-control analysis yielded the following results: (OR = 1.60, 95% CI: 1.28, 2.02, *p* = 0.933, I^2^ = 0%). As can be observed, the cohort study group has a high level of heterogeneity compared to the case-control study group, which has a low level. The significant degree of variation in the comprehensive results is thought to be caused, in part, by the type of study that was included in the literature.

#### 3.3.4. Subpopulation Analysis Based on PCB Type Was Conducted

**Figure 9:**
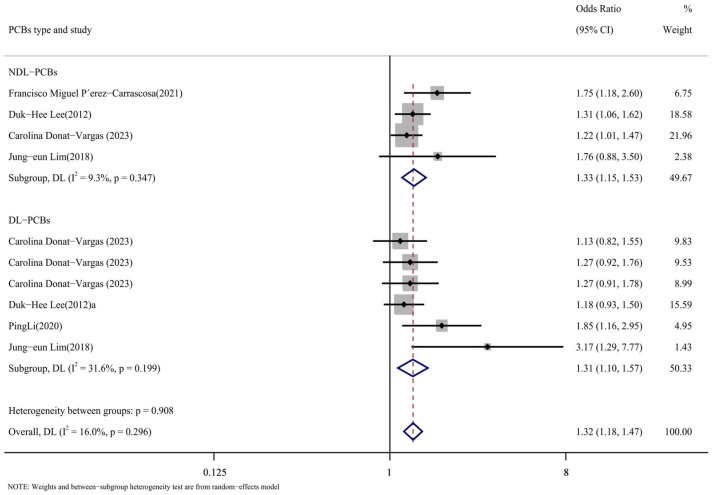
Forest map with PCB types as subgroup [24,26,27,28,29].

According to the types of PCBs, the literature was divided into the DL-PCB group [16,24,26,29] and the NDL-PCB group [24,26,28,29]. Figure 9 displays the outcomes of the subgroup analysis. The following were the NDL-PCB group’s findings: DL-PCB group results were as follows (OR = 1.31, 95% CI: 1.10, 1.57, *p* = 0.199, I^2^ = 31.6%; OR = 1.33, 95% CI: 1.15, 1.53, *p* = 0.347, I^2^ = 9.3%). The substantial heterogeneity of the comprehensive results cannot be attributed to the type of PCBs, as the heterogeneity of the DL-PCB group and the NDL-PCB group is both low and not significant. The findings suggested that there was a higher risk of cardiovascular illness associated with both DL-PCBs and NDL-PCBs.

In addition, we conducted a subpopulation analysis of different homologues of PCB types. As for the effects of different PCBs on cardiovascular diseases, four articles [16,24,27,29] reported PCB153, PCB138, and PCB180 for NDL-PCBs. Supplementary Figure S1 displays the findings for PCB153 (OR = 1.32, 95% CI: 1.19, 1.45, *p* = 0.411, I^2^ = 0%); PCB138 (OR = 1.19, 95% CI: 0.93, 1.54, *p* = 0.021, I^2^ = 69.2%); and PCB180 (OR = 1.09, 95% CI: 0.99, 1.19, *p* = 0.705, I^2^ = 0%). A strong conclusion was reached by using the fixed-effect model, which demonstrated a positive correlation between PCB153 and the incidence of cardiovascular illnesses without any heterogeneity.

This was followed by DL-PCBs, with three articles [24,26,29] reporting PCB118 and PCB156. Supplemental Materials Figure S2 displays the findings for PCB118 (OR = 1.26, 95% CI: 1.04, 1.52, *p* = 0.408, I^2^ = 1.2%) and PCB156 (OR = 1.18, 95% CI: 0.83, 1.67, *p* = 0.060, I^2^ = 52.8%). PCB118 was shown to have a significant increase in the risk of cardiovascular disease among them. The conclusion was supported by the low level of heterogeneity.

## 4. Discussion

We conducted a meta-analysis and systematic review to examine the link between cardiovascular disease and PCB exposure. We draw the conclusion that, due to its great heterogeneity, PCB exposure is a risk factor for cardiovascular disease based on a selection of 11 epidemiological studies conducted up until March 2024 among six nations. As a result, we performed subgroup analysis based on the biological material, research type, article quality, and PCB type. We discovered that these factors could be the cause of the article’s high heterogeneity.

The results of the subgroup analysis of PCB types for NDL-PCBs (OR = 1.33, 95% CI: 1.15, 1.53) and DL-PCBs (OR = 1.31, 95% CI: 1.10, 1.57) all indicated a low degree of heterogeneity in the positive association between PCBs and the risk of cardiovascular disease. NDL-PCB-153 (OR = 1.32, 95% CI: 1.19, 1.45) and DL-PCB-118 (OR = 1.26, 95% CI: 1.04, 1.52) were found to be risk variables for cardiovascular disease in homologous subgroup analysis. The E value of the PCB effect on cardiovascular disease was calculated in this study, and the result was 2.259, indicating a high degree of confidence and relative robustness. As a result, we think that our analysis’s findings are more precise and compelling.

The molecular pathways that could link PCBs to cardiovascular disease have been examined in earlier research. PCBs are well-known endocrine disruptors that mimic or obstruct the body’s hormonal functions, particularly those of the thyroid and sex hormones [31]. The changes in the metabolic rate induced by this disruption may affect the functioning of the cardiovascular system. For example, an imbalance in thyroid hormones can lead to abnormal blood pressure, altered cholesterol levels, and irregular heart rhythms, each of which is a known risk factor for cardiovascular disease [32]. Research has indicated that estrogen functions as a cardiac protective agent, regulating cardiovascular health and lowering blood pressure, vasoconstriction, vascular resistance, and heart hypertrophy [33]. These effects provide protection against cardiovascular disease, and an excess of estrogen may actually exacerbate the condition [33].

Chronic inflammation is closely linked to the onset of cardiovascular disease, particularly when atherosclerosis is forming. The growth and accumulation of plaque may be aided by the activation and deposition of inflammatory cells in the blood vessel wall [34]. The accumulation and activation of inflammatory cells in the vascular wall can promote the formation and progression of plaques. Dioxin-like PCBs (DL-PCBs) can initiate intracellular signaling by binding to the aryl hydrocarbon receptor (AHR), thereby activating inflammatory pathways in the body. For example, PCB126 induces inflammation by increasing the production of the inflammatory cytokine tumor necrosis factor-α (TNF-α) [35]. Studies have demonstrated that non-dioxin-like PCBs (NDL-PCBs) can stimulate the production of reactive oxygen species (ROS), leading to oxidative stress [36]. The oxidative stress induced by PCB118 may contribute to endothelial dysfunction, increased lipid peroxidation, and the degradation of nitric oxide in the endothelium, thereby impairing vascular relaxation and exacerbating hypertension and arteriosclerosis [36]. Furthermore, exposure to PCB153, a dioxin-like PCB (DL-PCB), may lead to the downregulation of lipoprotein lipase (LPL) activity, thereby affecting the hydrolysis and absorption of lipids. This, in turn, results in elevated levels of low-density lipoprotein (LDL) in the blood, thereby increasing the risk of cardiovascular disease [37].

The shortcomings of this meta-analysis are numerous. With only 11 studies included, it has a small sample size and low representativeness, which increases the risk of bias. Significant variability between the studies was also discovered in the analysis, with differing levels of research quality, measurement errors in exposure levels, and inadequate management of confounding variables. Generally speaking, a significant cause of heterogeneity is the variable quality of the included research. According to this research, high-quality studies could contain selection biases that impede their findings from accurately representing the target population’s real circumstances. Moreover, variations in sample size, follow-up duration, and data-processing techniques between the studies could impact the consistency and dependability of the findings. A dearth of high-caliber studies may also affect the results. The main focus of this investigation was on biological materials containing serum and plasma. Due to their high lipophilicity, PCBs usually attach to blood proteins. The changes in serum and plasma composition, especially the presence of coagulation factors, may have an impact on PCB binding status [38,39].

The action of coagulation factors may cause changes in plasma during storage, which could have an impact on PCB stability and measurement outcomes. Serum, on the other hand, remains reasonably stable following separation, which could lead to more accurate measurement results [40]. Cross-sectional, case-control, and cohort studies are the study types that are considered; an additional source of heterogeneity is the variations in these study designs. Subgroup analysis on case-control and cohort studies is the main focus of this research. Recall and selection bias are common problems in case-control research. On the other hand, longitudinal cohort studies, which frequently necessitate lengthy follow-up periods and substantial resources, are better able to evaluate the temporal association between exposure and disease. Moreover, a number of confounding variables, including nutrition, physical activity, education level, socioeconomic position, smoking, drinking alcohol, and exposure to other environmental contaminants, can affect the association between PCB exposure and cardiovascular disease. Variations in the degree to which these characteristics are adjusted in different studies may lead to biased findings. Furthermore, while examining the connection between PCBs and cardiovascular illness, it is important to consider the level of exposure, the length of exposure, and the relevant lifetime periods of sensitivity.

## 5. Conclusions

The connection between PCB exposure and the risk of CVDs was thoroughly assessed in this meta-analysis, which found that exposure to PCBs considerably raises the risk of CVDs. This discovery offers crucial scientific support for our comprehension of how environmental contaminants affect human health. Despite certain methodological limitations, the study’s findings generally show a favorable association between exposure to PCBs and cardiovascular diseases. The current findings provide preliminary evidence for a potential association between PCBs and cardiovascular disease. However, this field still requires more detailed studies to further elucidate the underlying mechanisms and the diversity of effects. In conclusion, this study emphasizes the significance of lowering exposure to PCBs and offers a solid scientific foundation for therapeutic and public health actions. It highlights the need for individuals and society to take measures to reduce PCB exposure in order to lower the risk of CVDs.

## Figures and Tables

**Table 1:** Main features of the included literature.

Reference	Location	Study Design	Study Population	Time- Period	Sample Size	Age (Year)	Ratio of Sex	Biomaterials	PCBs Type	Method of Measurement	Results ^a^	Main Finding
Charlotte Bergkvist et al. (2016) [14]	Swedish	Cohort study	Lived in Central Sweden and did not have cardiovascular disease.	1998–2009	36,759	45–79	All male	Serum	6 PCB congeners	Unclear	ΣPCBs OR 1.97 95% CI [1.42–2.75)] ^b^	Exposure to PCBs via diet was associated with increased risk of myocardial infarction in men.
Carolina Donat-Vargas et al. (2020) [15]	Aragon	Cross-sectional	Men without cardiovascular disease enrolled in the Aragon Workers’ Health Study	2011–2014	1844	40–60	All male	Serum	4 PCB Congeners	Computerized tomography	ΣPCBs OR 2.03, 95% CI [1.21–3.40]	PCB exposure seems to increase the risk of coronary disease in men from the very early stages.
Francisco Miguel P´erez-Carrascosa et al. (2021) [16]	Spanish	Cohort study	Participants were recruited in two hospitals in Granada province, Southern Spain.	15-year follow-up study	387	Unclear	Unclear	Adipose tissue	3 PCB congeners	Gas chromatography coupled to mass spectrometry in tandem mode	Main PCB138 OR 1.75, 95% CI [1.18–2.60]	Long-term PCB exposure might represent a modifiable risk factor for CVD.
Laura Deen et al. (2023) [17]	Danish	Cohort study	The HESPAIR cohort includes all residents of two residential areas in the Greater Copenhagen area	21-year follow-up study	51,249	Children and adults	Unclear	Plasma	7 PCB congeners	Unclear	ΣPCBs HR 1.02, 95% CI [0.94–1.10]	Cumulative residential exposure to airborne PCB was not associated with a higher overall risk for CVD.
Duk-Hee Lee et al. (2012) [24]	Swedish	Cohort study	Study subjects at baseline were 1016 subjects who were 70 years old and residents of Uppsala	5-year follow-up study	898	70	Unclear	Plasma	16 PCB congeners	HRGC/HRMS	ΣPCBs OR 5.5 95% CI [1.7–18.1]	Background exposure to POPs may play an important role in development or progression of stroke in the elderly.
Charlotte Bergkvist et al. (2014) [25]	Swedish	Cohort study	Women residing in central Sweden	12-year follow-up study	34,591	Unclear	All women	Serum	6 PCBs	Unclear	ΣPCBs OR 1.65; 95% CI [1.27–2.15]	Dietary exposure to PCBs was associated with an increased stroke risk in women, especially haemorrhagic stroke.
Jung-eun Lim et al. (2018) [26]	Korean	Case-control study	Participants in routine health assessment at 11 health promotion centers in Seoul and Gyeonggi Province	2004–2013	637	>20	Unclear	Serum	32 PCBs	GC/HRMS	ΣPCBs OR 1.66; 95% CI [0.8–3.45] ΣDL-PCBs OR: 3.17; 95% CI [1.29–7.77] ΣNDL-PCBs OR: 1.76; 95% CI [0.88–3.5]	Elevated serum POPs levels were associated with an increased risk of stroke, especially ischemic stroke.
Elena Raffetti et al. (2018) [27]	North Italy	Cohort study	Urban residents near chemical plants that produce PCBs	2001–2013	1331	Mean age 50.6 years	45.7% males and 54.3% women	Serum	24 PCBs	Hewlett–Packard 6890 N gas chromatograph coupled with an MSD HP 5973.	ΣPCBs RR 2.07, 95% CI [1.18–3.63]	These results suggest that PCBs might play a role in the development of possibly cardiovascular disease, though alternative explanations are to be considered, too.
PingLi et al. (2020) [28]	China	Case-control study	Stroke Patients and Healthy Population Recruited at Tianjin Hospital, China	2017–2019	482	>20	56% males and 44% women	Serum	17 PCBs	GC/MS	ΣPCBs OR 1.704; 95% CI [1.073–2.506] ΣDL-PCBs OR: 1.846; 95% CI [1.156–2.949]	The results suggest detrimental roles of PCBs, mainly dioxin-like PCBs, in stroke risk, irrespective of stroke subtypes.
Carolina Donat-Vargas et al. (2023) [29]	Swedish	Cohort study	The SMC included women born from 1914 to 1948 residing in central Sweden	Mean age 61: 15-year follow-up study Mean age	1528	Mean age 61	33% males and 67% women	Blood lipids	12 PCBs	Gas chromatography–triple quadrupole mass spectrometry	ΣPCBs OR 1.28; 95% CI, [0.92–1.79]	Dioxin-like PCBs and non-dioxin- like PCBs were significantly associated with cardiovascular disease.
Tessa Schillemans et al. (2024) [30]	Swedish	Nested case-control design	The study used data from the SMC-C, which is part of the Swedish Infrastructure for Medical Population-Based Life-Course and Environmental Research	2003–2009	657	<85	All Women	Plasma	13 PCB congeners	GC-MS/MS	ΣPCBs OR 1.55; 95% CI [1.16–2.09]	PCBs correlated positively with omics associated with increased MI and stroke risk.

^a^ The estimation is presented for the highest category compared with the lowest for the most adjusted model. ^b^ Estimated full intake.

## Data Availability

The data that support the findings of this study are available from the corresponding author upon reasonable request.

## Supplementary Materials

The following supporting information can be downloaded at: https://www.mdpi.com/article/10.3390/toxics13020071/s1, Search term; Table S1: Control for confounding; Table S2: Egger’s test of total PCBs and CVDs; Figure S1: Forest map with NDL-PCB homolog as a grouping; Figure S2: Forest map featuring DL-PCB homolog as a distinct subgroup.
