# Supplementary material for: Uncovering the Potential Link Between Polychlorinated Biphenyls and Cardiovascular Diseases: A Comprehensive Analysis
Source: Toxics. 2025 Jan 22;13(2):71. doi: 10.3390/toxics13020071 (PMC11860408; doi:10.3390/toxics13020071)
Supplement: Supplementary file 1 [file toxics-13-00071-s001.zip › toxics-3409569-supplementary.pdf]

Suppementary Matetials: search term

We utilized certain reference terms, with an example in PubMed as follows:

(((Polychlorinated Biphenyl) OR (PCBs)) OR (Polychlorobiphenyl Compounds)) AND ((Cardiovascular Disease) OR (Cardiovascular Abnormalities)) OR (Heart Diseases)) OR (Cardiomyopathies)) OR (Vascular Diseases)) OR (Angioedema)) OR (Venous Insufficiency)) OR (Hemorrhoids)) OR (Myocardial infarction)) OR (apoplexy))

We utilized certain reference terms, with an example in Web of Science as follows:

((TS=(Cardiovascular Disease)) OR TS=(Cardiovascular)) OR TS=(Vascular Malf ormations)) OR TS=(Cardiomegaly)) OR TS=(Heart Neoplasms)) OR TS=(Peric arditis)) OR TS=(Aortic Diseases)) OR TS=(apoplexy)) OR TS=(Myocardial in farction)) OR TS=(Reperfusion Injury)) OR TS=(Telangiectasis) AND ((TS=(PC Bs)) OR TS=(Polychlorinated biphenyls)) OR TS=(Polychlorobiphenyl Compoun ds)

We utilized certain reference terms, with an example in Cochrane Library as follows:

(Polychlorinated biphenyls AND cardiovascular diseases OR PCBs AND heart disease OR PCBs AND stroke OR Polychlorinated biphenyls AND atherosclerosis OR Polychlorinated biphenyls AND hypertension OR PCBs AND myocardial infarction OR PCBs AND CVD risk OR PCBs exposure AND cardiovascular health OR Environmental pollutants AND cardiovascular disease OR Persistent organic pollutants AND heart disease Polychlorinated biphenyls AND cardiovascular effects OR PCBs exposure AND vascular disease OR Environmental exposure to PCBs AND cardiovascular outcomes OR PCBs bioaccumulation AND cardiovascular impact)

We utilized certain reference terms, with an example in ScienceDirect as follows:

“Polychlorinated Biphenyl” OR “Cardiovascular Disease”

Supplementary Table 2 Egger's test of total PCBs and CVDs

Number of studies = 11 Root MSE = .8957

Std_Eff	Coef.	Std. Err.	t	P>|t|	[95% Conf. Interval]	
slope	-.0855986	.0444037	-1.93	0.086	-.1860467	.0148496
bias	3.239333	.4267393	7.59	0.000	2.273982	4.204685

Test of H0: no small-study effects P = 0.000

Supplementary Fig 1: Forest map with NDL-PCB homolog as a grouping.

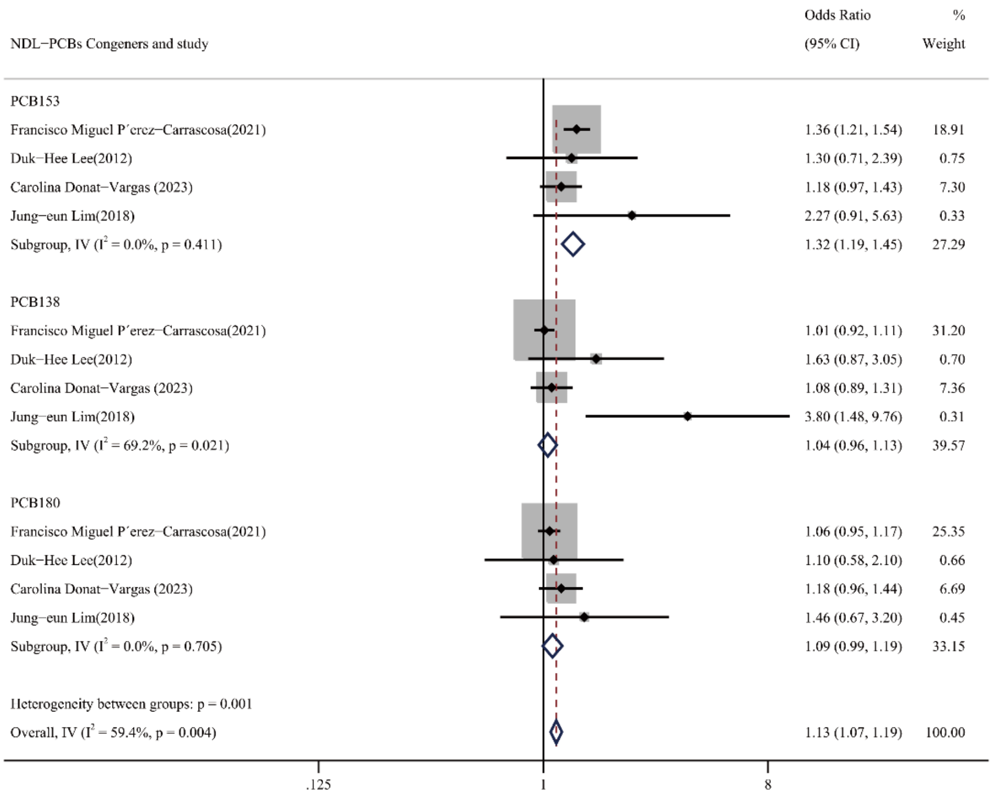

Supplementary Fig 2: Forest map featuring DL-PCB homolog as a distinct subgroup

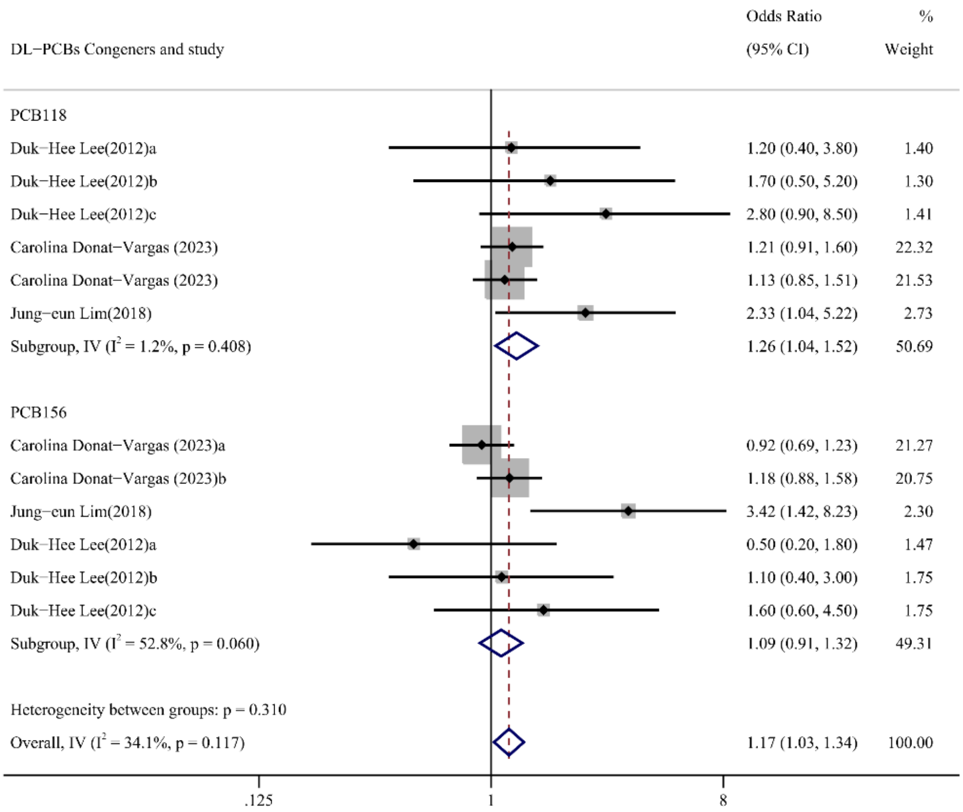

Supplementary Table 1. Control for confounding.

Study: first author and year of publication	Age	Sex	Body mass index	Smoking habit	Tipple	Physical activity	Diabetes	Educational level	Family history of disease	Triglycerides and cholesterol
Tessa Schillemans et al. 2024	Yes	Yes	Yes	Yes		Yes	Yes	Yes	Yes	Yes
Laura Deen et al. 2023	Yes	Yes						Yes		
Francisco Miguel Perez-Carrascosa et al. 2021	Yes	Yes	Yes	Yes	Yes			Yes		
Elena Raffetti et al.2018	Yes	Yes	Yes	Yes	Yes			Yes		Yes
Duk-Hee Lee et al. 2012		Yes	Yes	Yes	Yes	Yes	Yes			Yes
Charlotte Bergkvist et al. 2016	Yes			Yes	Yes	Yes		Yes	Yes	Yes
Carolina-Donat–Vargas et al. 2023	Yes	Yes	Yes	Yes		Yes		Yes	Yes	
Carolina-Donat–Vargas et al. 2020	Yes		Yes	Yes	Yes	Yes	Yes	Yes		Yes
Ping Li et al.2021	Yes		Yes	Yes	Yes	Yes	Yes	Yes		Yes
C Bergkvist et al.2014	Yes		Yes	Yes	Yes			Yes	Yes	Yes
Jung-eun Lim et al.2018	Yes	Yes	Yes	Yes	Yes	Yes			Yes	Yes
